# The Tervuren xylarium Wood Density Database (TWDD)

**DOI:** 10.1038/s41597-026-06563-2

**Published:** 2026-01-16

**Authors:** William W. M. Verbiest, Pauline Hicter, Hans Beeckman, Daniel Wallenus, Bhély Angoboy Ilondea, Jean-François Bastin, Marijn Bauters, Jérôme Chave, Ruben De Blaere, Thalès de Hauleville, Tom De Mil, Maaike de Ridder, Cécile De Troyer, Corneille E. N. Ewango, Adeline Fayolle, Anais Gorel, Fabian Jörg Fischer, Begüm Kaçamak, Christien Kimbuluma, Nestor K. Luambua, Félix Laurent, Kévin Liévens, Jean-Remy Makana, François Malaisse, Mbusa Wasukundi, Michael Monnoye, Alfred Ngomanda, Franck Rodrigue Olouo Ambounda, Benjamin Toirambe, Cédric Otepa, Joris Van Acker, Bes Van den Abbeele, Jan Van den Bulcke, Blanca Van Houtte Alonso, Thierry Wankana, Brice Yannick Djiofack, Wannes Hubau

**Affiliations:** 1 Service of Wood Biology, Royal Museum for Central Africa, Tervuren, Belgium; 2 Department of Environment, Laboratory of Wood Technology (Woodlab), Ghent University, Ghent, Belgium; 3 Department of Environment, Q-ForestLab, Ghent University, Ghent, Belgium; 4 Wood Laboratory of Yangambi, Institut National pour l’Étude et la Recherche Agronomiques, Yangambi, Democratic Republic of Congo; 5 Université Pédagogique Nationale, BP 8815 Kinshasa-Ngaliema, Democratic Republic of Congo; 6 Cerac.be, Avenue Galilée, 5/2, BE 1210 Brussels, Belgium; 7 TERRA Teaching and Research Centre (Forest is Life), Axe de Gestion des Ressources Forestières, Gembloux Agro-Bio Tech, University of Liege, Passage des Déportés n°2, 5030 Gembloux, Belgium; 8 Laboratoire Evolution et Diversité Biologique, CNRS and Université Paul Sabatier, UMR 5174 EDB, Toulouse, 31000 France; 9 Faculté de Gestion de Ressources Naturelles Renouvelables, Université de Kisangani, Kisangani, Democratic Republic of Congo; 10 CIRAD, Forest and Societies research unit, Montpellier, France; 11 School of Biological Sciences, University of Bristol, Bristol, BS8 1 T United Kingdom; 12 Faculté des Sciences, Laboratoire d’Écologie et Aménagement Forestier, Université de Kisangani, Kisangani, Democratic Republic of Congo; 13 Biodiversity, Ecosystems, Landscapes, University of Liège—Gembloux Agro-BioTech, 5030 Gembloux, Belgium; 14 Ecole Régionale Post Universitaire d’Aménagement et de Gestion Intégrés des Forêts et Territoires Tropicaux, Kinshasa, DRC Democratic Republic of the Congo; 15 Faculty of Agricultural Sciences and Environment, Department of Renewable Natural Resources Management, Université Catholique du Graben, Butembo, DRC Democratic Republic of the Congo; 16 Centre National de la Recherche Scientifique et Technologique (CENAREST), Libreville, Gabon; 17 Université des Sciences et Techniques de Masuku, Franceville, Gabon; 18 Ministère de l’Environnement et Développement Durable, Kinshasa, Democratic Republic of the Congo

**Keywords:** Forestry, Forest ecology, Tropical ecology, Carbon cycle, Forest ecology

## Abstract

Wood density is a key plant property, indispensable for estimating forest biomass. Yet, despite tropical regions’ substantial contributions to global tree diversity and carbon cycling, they remain underrepresented in wood density datasets such as the CIRAD and Global Wood Density Database (GWDD). To address this gap, we present the ‘Tervuren xylarium Wood Density Database’ (TWDD), containing 13,332 samples from 2,994 species, 1,022 genera, and 156 plant families across six continents (72% from Africa). TWDD offers direct measurements of oven-dry (oven-dry mass/oven-dry volume, all samples), air-dry (air-dry mass/air-dry volume, 6,408 samples), green (green mass/green volume, 1,657 samples), and basic wood density (oven-dry mass/green volume, 1,686 samples). Basic density was estimated for the remaining 11,646 samples via conversion from oven-dry density. TWDD closes a substantial wood density data gap, especially in Africa, adding 1,164 new species, 160 new genera, and 8 new plant families not included in GWDD or CIRAD datasets. The TWDD provides a critical resource for advancing research on forest community dynamics, ecosystem functioning, carbon cycling, and trait-based ecology worldwide.

## Background

A key metric to estimate dry biomass and carbon content of woody vegetation is the “basic wood density”, defined as oven-dry mass divided by green volume. Green woody volume is typically measured through dendrometry^1–4^ (or sometimes terrestrial LiDAR^5–7^) and converted to (oven-dry) biomass through multiplication with basic wood density. Wood density is also an important summary trait that aggregates various wood morphological traits (e.g., conduit diameter, lignin content, ground tissue cell-wall thickness, extractives, etc.)^4,8,9^ and reflects trade-offs in tree structure, carbon storage, hydraulics, drought tolerance, and demographics (i.e., rates of mortality, recruitment, and growth)^4,8,10–25^. Therefore, large global datasets of basic wood density emerged such as the CIRAD^2,26^ and Global Wood Density Database (GWDD)^4,9^. Hyperdiverse tropical regions are generally underrepresented in these datasets, particularly Africa^27^ with only 3,077 samples in the GWDD^4,9^ (19% of dataset) and 8,798 samples in the CIRAD dataset^2,26^ (37% of dataset). In tropical Africa, 9,514 tree species are currently documented^28^, from which the GWDD^4,9^ and CIRAD datasets^2,26^ only cover about 11% and 18%, respectively.

The scarcity of wood density data from Afrotropical forests is problematic since these ecosystems are crucial regulators of global carbon cycling^23^ Wood density data is used in combination with data from repeated forest inventories to quantify tropical forest carbon stocks, carbon gains, losses, and net fluxes^22–24,29–38^. These analyses revealed that African tropical forests are a large, stable carbon sink, which had major scientific and policy impact^23^. Inventory datasets are being expanded and are regularly used to monitor and predict responses of forest carbon fluxes to environmental changes^15,39–45^. However, surprisingly little efforts have been made to expand wood density datasets in tropical Africa, leading to increasing mismatches between inventory and wood density data.

Basic wood density values in available datasets are mostly derived from air-dry or oven-dry wood density measurements, which are converted to basic wood density using conversion factors calculated using wood samples with simultaneous green and air-dry and/or oven-dry wood density measurements^1,2,4,46–48^, each reflecting a different moisture state of wood^2,49^. Green wood density is the mass-to-volume ratio of freshly collected wood from a living tree, measured immediately in the field after sampling. Air-dry wood density is air-dry wood mass divided by air-dry volume, typically reflecting wood at approximately 12% moisture in ambient climatic conditions in temperate regions^2^. Oven-dry wood density is oven-dry mass divided by oven-dry volume, representing wood density at 0% moisture (anhydrous state)^49^. However, these three metrics face several limitations.

Green wood density is seldomly recorded, because it requires immediate processing after sampling, which is often impractical during field campaigns^1,2^. Some studies determine it by re-immersing wood samples in water in the lab for several days, likely misrepresenting natural conditions of green wood. Air-dry wood density is challenging to standardize due to variable moisture content of wood (typically 8–18% depending on storage conditions^50^), limiting comparability across wood density datasets^2,49^. In contrast, oven-dry wood density has a clearer definition with international standards (e.g., ISO-13061-1:2014)^51^ and classic wood references^52–56^ prescribing a drying temperature of 103 ± 2 °C with drying time determined during the drying process^51^ and gradual temperatures increases to prevent damage^2^. However, oven-drying protocols vary between studies^1,12,19,46,49,57–66^, particularly in oven-drying duration due to factors such as the varying sizes and density of the wood samples^67^. Consequently, methodologies for estimating wood density are often unclear, especially regarding drying procedures and whether basic density was derived from air- or oven-dry wood density.

The Tervuren xylarium, established in 1898 by Belgium’s Royal Museum for Central Africa (RMCA), was originally created to demonstrate the economic value of African tropical timber^26^. Its focus later expanded to include scientific research on both commercial and non-commercial tropical African tree species^68–70^. Since the mid-20^th^ century, the collection has also incorporated specimens from other continents. Today, it is Belgium’s main scientific wood reference collection, comprising over 83,000 specimens from 13,533 species and lower taxa (about one-third from Africa). Over 30% of the collection or 26,604 specimens originate from the Democratic Republic of the Congo (DR Congo), representing more than 2,000 woody species and lower taxa, including timber trees, small trees, shrubs, dwarf shrubs, and lianas. 6,953 specimens are paired with herbarium samples^71^. The collection supports reference databases for wood anatomy and identification using image recognition^72^ or chemical fingerprinting^73,74^. Applications include law enforcement (ENFORCE: https://enforce.africamuseum.be/en), cultural heritage^75^, archaeobotanical^76^ or palaeoecological studies^77^, as well as research in archaeology, timber quality assessment, forest ecology^78^, wood technology^73^, and dendrochronology^79^. Consequently, the large Tervuren wood collection makes it a key source for generating a new basic wood density dataset, especially for the underrepresented African tropics.

Here, we present the ‘Tervuren xylarium Wood Density Database’ (TWDD), which is not integrated in the CIRAD^2,26^ or GWDD databases^4,9^ yet, and has a strong focus on tropical Africa. The TWDD represents 13,332 wood samples (of which 9,601 samples or 72% of the whole dataset from Africa) for which air-dry (for 6,408 samples or 48% of data) and oven-dry wood density (for all samples) were measured using clear, consistently applied protocols. For 1,657 wood samples, green wood density was also measured in the field on the same day of sampling. Additionally, we used the TWDD to produce new conversion factors to calculate basic wood density from air-dry and oven-dry wood density for African tree species. Using the TWDD, we addressed three research questions attempting to clarify a few commonly held assumptions about wood density:

1. What is the ideal oven-drying time for air-dried wood samples in the Tervuren xylarium?
2. Do our new African conversion factors to compute basic wood density from air-dry and oven-dry wood density corroborate previously published (global) conversion factors^1,2,4,46–48^?
3. Do species-level average basic wood densities differ between the TWDD versus the GWDD^4,9^ and CIRAD databases^2,26^?

Although building on previous protocols^2,4,9,46,50,51^, our study clarifies previous methodological caveats regarding quantifying wood density metrics using a clear, well-defined standard protocol, which can be used to standardize the creation of large wood density datasets.

## Methods

### Air-dry wood density measurements

Wood specimens collected in the 19^th^ and 20^th^ century were directly stored in the xylarium and left to equilibrate with ambient (temperate) climatic conditions. Since 2005, newly collected samples were oven-dried progressively from 30 to 70 °C during 1–4 days before storing them in the Tervuren xylarium, to avoid contamination by insects or fungi by reaching a moisture content of less than 20%^80^. They were then left to equilibrate in the xylarium at ambient climatic conditions, with a relative air humidity around 40% and air temperatures approximating 20 °C. Measurement of air-dry and oven-dry wood density was initiated in 2010 using a well-defined wood density measurement protocol^67^. We systematically measured newly collected samples, but also older samples that were used for specific projects such as SmartWoodID^72^ and HerbaXylaRedd (Home | herbaxylaredd.africamuseum.be)^71^.

**Fig. 1:**
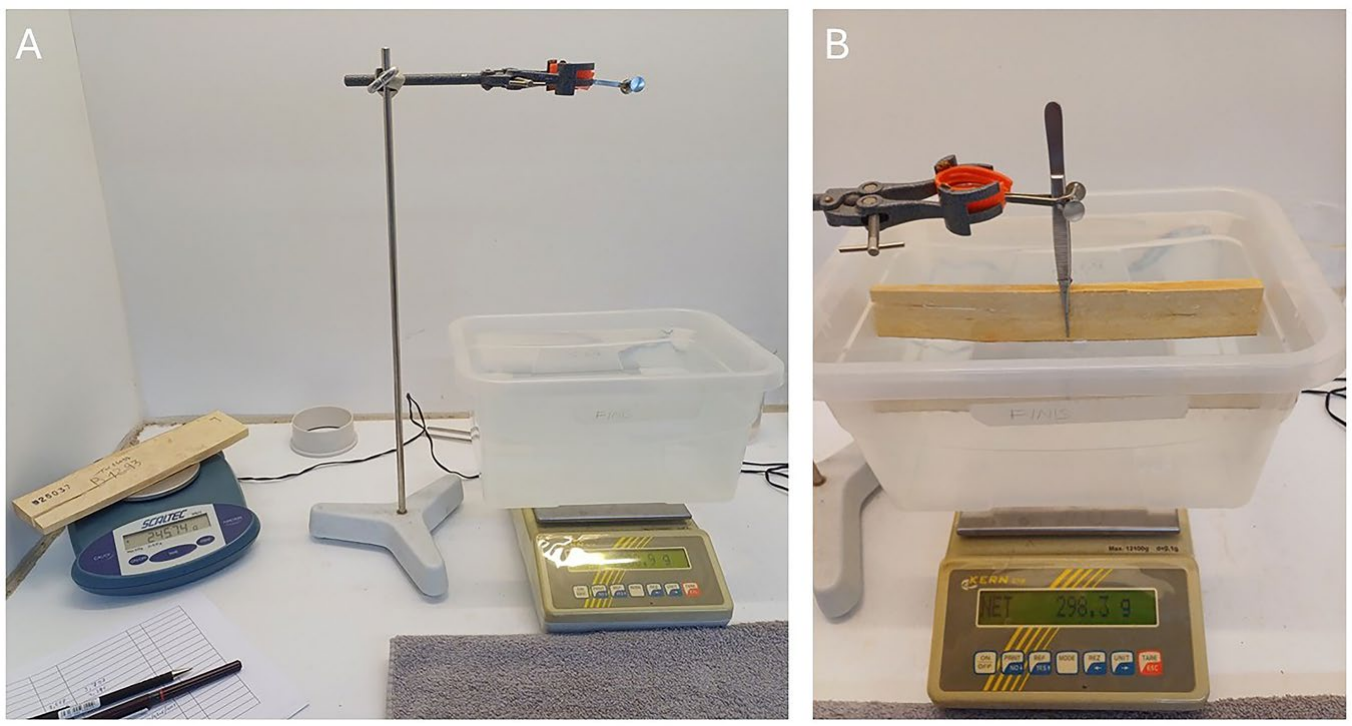
Overview of methods to measure wood mass and volume for small wood samples. Set-up for wood density measurements of small samples. Mass is measured using a balance with a precision of 0.01 g (**A**, on the left side). Volume is measured using the water displacement method by submerging the sample in a small water container with tweezers on a balance with a precision of 0.1 g (**B**).

**Fig. 2:**
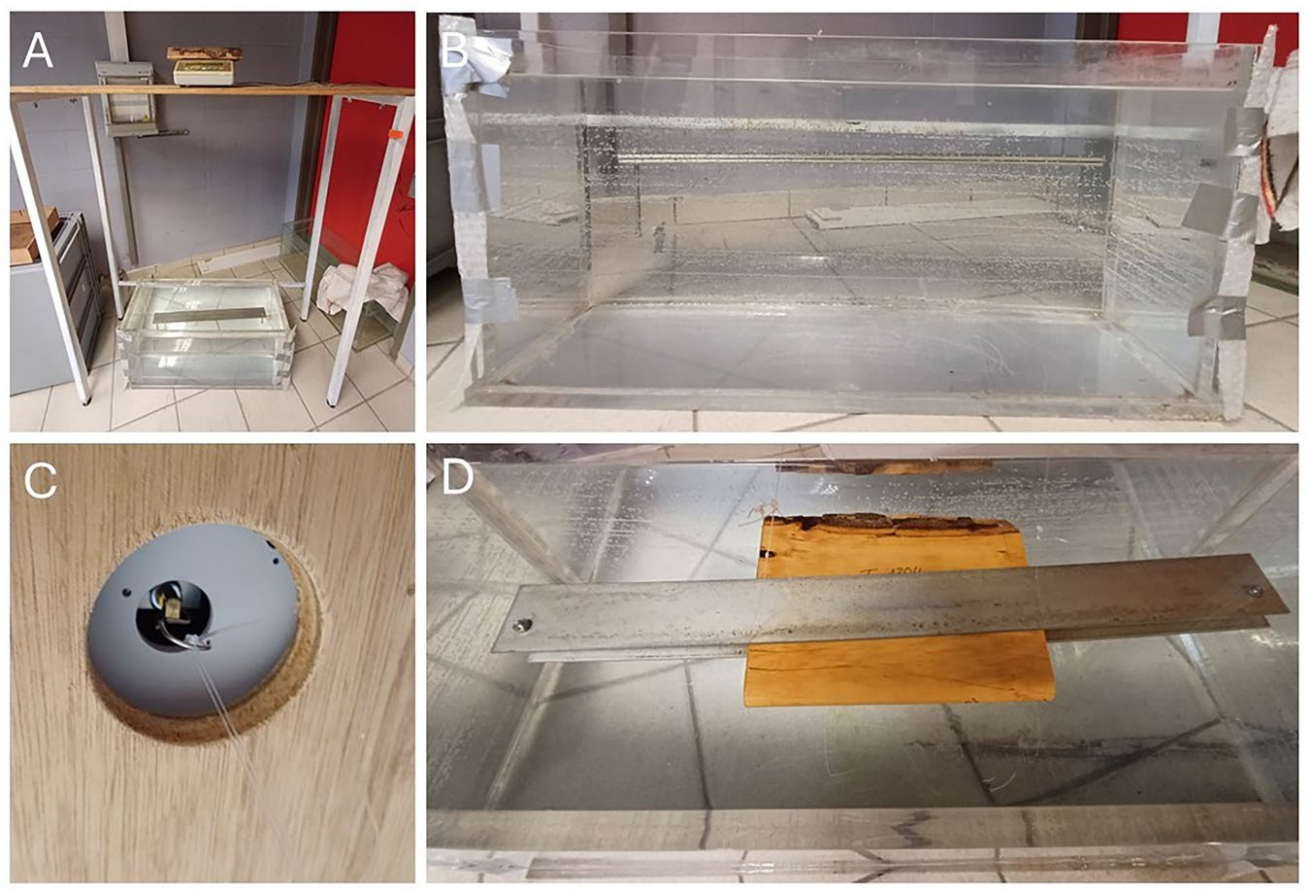
Overview of methods to measure wood mass and volume for large samples. Set-up for wood density measurements of large samples (old samples in the collection) (**A**). Mass is measured using a precision balance of 0.1 g (located on the shelft at the top of image A). Volume is measured by submerging the sample which is clamped between two metal plates in a large water container (**B**) attached to a balance with precision 0.1 g using a plastic wire (**C-D**). The volume of the metal plates is subtracted from the total measured volume to calculate the volume of the wood.

We measured air-dry mass (expressed in g) and volume (expressed in cm^3^). Air-dry mass was measured using a balance with a precision of 0.01 g and a maximum weighing capacity of 610 g. Air-dry mass of samples greater than 610 g was measured using a balance with a precision of 0.1 g. Volume was measured by submerging the sample using tweezers in a plastic container (height 12.5 cm, length 26 cm, and width 20 cm) filled with distilled water on a balance with a precision of 0.1 g (see above), using the water displacement method following Archimedes’ principle^57^ (Fig. 1). Volume of samples larger than the dimensions of the container or thicker than 3 cm (limited by tweezer size) were measured by submerging samples clamped between a metal rod attached to a balance with a precision of 0.1 g in a large water container (height 39 cm, length 82 cm, and width 33 cm) (Fig. 2). The volume of the tweezers was subtracted from the total measured volume to calculate the volume of the wood. Air-dry wood density was then calculated as air-dry mass divided by air-dry volume (expressed in g cm^−3^).

### Oven-dry wood density measurements

Samples were then put in a drying oven at 103 ± 2 °C for 24 h. To prevent damage to the wood samples due to rapid thermal expansion, we gradually increased the temperature in the oven from 60 °C (during 2 h) to 80 °C (during 4 h) and eventually 103 °C (during the remaining 18 h). Oven-dry mass and volume were then measured immediately after drying using the same balances and setups as for air-dry measurements. Each time, we took five samples from the oven to measure wood density, while leaving the other samples in the oven to avoid uptake of moisture. The time between the first and last wood sample measurements dried in the oven ranged between 5 and 6 h. Oven-dry wood density was then calculated as oven-dry mass divided by oven-dry volume (expressed in g cm^−3^). We repeated wood density measurements of samples when wood density was higher than 2 g cm^−3^ to correct potential anomalous outliers.

### Air-dry moisture content

To evaluate air-dry conditions of wood samples in the Tervuren xylarium, we quantified air-dry moisture content (WC; expressed in %) of all wood samples using wood mass at air-dry (${{\rm{m}}}_{{\rm{air\; dry}}}$) and oven-dry conditions (${{\rm{m}}}_{{\rm{oven\; dry}}}$) (Eq. 1)^81^. In case samples had high, anomalous air-dry moisture content values (i.e., greater than 20%), we repeated oven-drying and remeasured the mass and volume of these samples to verify our moisture content estimates.

1 $${\rm{WC}}=\frac{{{\rm{m}}}_{{\rm{air\; dry}}}-{{\rm{m}}}_{{\rm{oven\; dry}}}}{{{\rm{m}}}_{{\rm{oven\; dry}}}}\ast 100\,[ \% ]$$

### Oven-drying time experiment

Oven-drying time affects wood density measurements by influencing removal of free water in cell cavities and other voids, as well as cell-bound water interacting with lignin and cellulose in the cell walls, with outcomes varying by wood density and volume. Depending on the size, density or composition of the different anatomical tissues (vessels, rays, parenchyma), higher-density wood often contains less water, but potentially requires longer oven-drying to fully remove all water^8,9,60,82^. Similarly, large-volume samples may need more time due to containing more water, while water in the sample’s center takes longer to be removed^60,82^. As such, to assess the minimum time required for completely oven-drying wood samples to quantify oven-dry wood density, we randomly selected 40 wood samples from the Tervuren xylarium in four main categories. The grouping was based on the 2.5–17.5% (low) and 82.5–97.5% (high) quantiles of both oven-dry wood density (0.347–1.043 g cm^−3^) and volume (17.8–428.3 cm^−3^): (1) high volume and high density, (2) high volume and low density, (3) low volume and high density, and (4) low volume and low density.

We measured air-dry wood mass, volume, and density as described above. Subsequently, we oven-dried samples at 103 °C for 24 h according to the protocol described above to measure oven-dry wood mass, volume, and density. After acclimating to the air-dry conditions of the Tervuren xylarium for a minimum of three months, we measured oven-dry mass and volume to compute density of wood dried in the oven for 48 h (2 h at 60 °C, 4 h at 80 °C, and 42 h at 103 °C). We do not account for potential hysteresis effects of oven-drying on wood density since they are thought to be limited^83^.

To test differences between wood mass, volume, and density between the four categories, we compared wood mass, volume, and density using violin plots and a Wilcoxon test using the function ‘wilcox.test’ from R package *stats*^84^. Furthermore, we quantified the Root Mean Square Error (RMSE) using function ‘rmse’ in R package *Metrics*^85^.

### Green wood density measurements

**Fig. 3:**
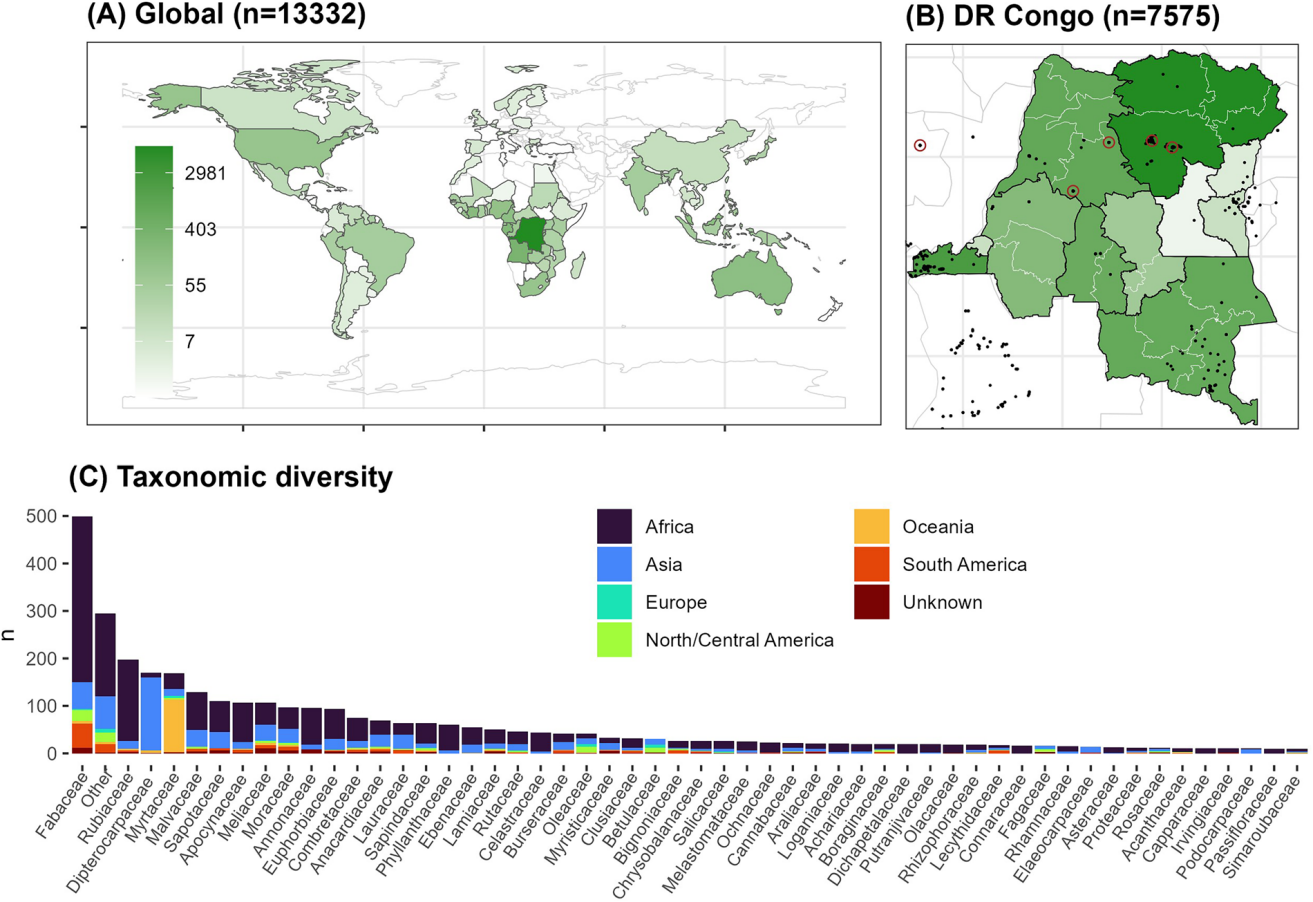
Geographic and taxonomic coverage of the Tervuren xylarium Wood Density Database (TWDD). Global coverage (**A**) and coverage per grouped province in the Democratic Republic of the Congo (DR Congo) (**B**). Grouped provinces of DR Congo are shown by the black lines, whereas present provinces are shown by the light-grey lines. The number of wood samples per country (panel A) and grouped province (panel B) (n) is visualized by the color, using a log-scale (see legend on the left panel). The total sample size is shown at the top of each panel. The country is unknown for 2,682 wood samples. 321 wood samples were not assigned to a province in DR Congo. Black points in panel B indicate wood samples with GPS coordinates. The five sites with green wood density measurements are indicated with brown circles in panel B. (**C**) Taxonomic diversity shown as the number of species per plant family (n, as bars). Bar colors indicate the sampling region (see legend on the right). Plant families with less than ten species are combined in one group called ‘Other’. See the **Overview Table** in Excel sheet of the TWDD for an overview of the regional geographic and taxonomic coverage of the data.

To establish factors to convert air-dry and oven-dry wood density into basic wood density for (tropical) African tree species, we conducted green wood density measurements of outer wood at five locations scattered across the region (i.e., Salonga National Park, Djolu, the Yangambi Biosphere Reserve, the Babagulu reserve, and the Ipassa research station) and two countries (i.e., the Democratic Republic of the Congo and Gabon, see Fig. 3 below). Using forest plot inventory data^24,25,86^, we selected dominant tree species by determining a representative set of species covering a minimum of 70% of basal area and stem number at each study site^87–89^.

In the field, we sampled at least three individuals per dominant tree species per site (except in Ipassa due to time constraints). To standardize wood density measurements and prevent any effects due to age differences, we only sampled mature, large, sun-lit trees reaching the canopy and with a diameter-at-breast-height ≥20 cm^57^. From each tree, we collected two outer wood samples at a sampling height of 1.1 m above the soil using an electric drill device with a dendrochronological borer (length 30 cm and diameter 1.2 cm). In case of absence of electricity (e.g., Salonga National Park), we used a manual hand crank drill attached to a cylinder saw (length 3.8 cm and diameter 2.2 cm) using an adaptor. We always sampled wood cores 30 cm above deformations or buttresses. If needed, we used an extendable telescope ladder to reach the sampling location. During the same day of sampling, we then measured green wood mass using a field balance with a precision of 0.01 g, and green wood volume via submersion. Green wood density was then calculated as green mass divided by green volume (expressed in g cm^−³^).

### Conversion factors to compute basic wood density for African tree species

We shipped the samples used for green wood density to the Tervuren xylarium and measured air-dry and oven-dry wood density as described above (after oven-drying for 3 days at 40 °C and air-drying for 2-3 months). This also allowed calculating air-dry moisture content (see above) and basic wood density (oven-dry mass divided by green wood volume).

To compute factors to convert air and oven-dry to basic wood densities for African tree species, we estimated the Pearson correlation coefficient between air-dry and basic wood density, and between oven-dry and basic wood density for all wood samples with density measurements at green, air-dry, and oven-dry conditions^2^. All density measurements were expressed in the same units and subject to the same types and size of errors. As such, we used type II regression models (i.e., Major Axis regression (MA)) for quantifying the slope of the symmetric relationship using the function ‘ma’ from R package *smatr*^90^, with an intercept forced through the origin. We conducted a similar analysis using species-level averages of air-dry, oven-dry, and basic wood density. All statistical analyses were performed in R v4.4.0^84^.

### Statistical analysis and dataset curation

To quantify basic wood density for wood samples without green wood volume measurements (87.4% of TWDD dataset), we used factors to convert oven-dry wood density to basic wood density for African tree species (59.4% of TWDD dataset) from our own study and for specimens sampled elsewhere (28% of TWDD dataset) from Vieilledent *et al*.^2^. We decided to use the oven-dry instead of air-dry to basic wood density conversion factor because air-dry moisture content differs among collections, with below-average moisture content in the TWDD.

Taxonomic names in the TWDD, GWDD and CIRAD datasets were corrected using the ‘WFO.match’ function in R package *WorldFlora* version June 2024^91,92^. From the TWDD, we excluded samples with unknown family names or unmatched species or genus names with the World Flora Online (WFO) dataset of plant taxonomic names^93^. To compare basic wood density estimates among datasets, we excluded samples with unknown genus or species. Thereafter, we calculated the Pearson correlation coefficient and performed MA regression (intercept not forced through zero)^90^, linking species-level average basic wood densities from TWDD with the GWDD^4,9^ and CIRAD datasets^2,26^ using R package *stats*^84^.

To test the statistical difference between the species-level average basic wood density from the TWDD and the other two datasets, we then performed a Wilcoxon test using the function ‘wilcox.test’ from R package *stats*^84^. Furthermore, to test the average difference between datasets, we quantified the Root Mean Square Error (RMSE) based on the species-level average wood density of the TWDD versus the CIRAD and GWDD datasets using the function ‘rmse’ in R package *Metrics*^85^. Additionally, we calculated the average percentage difference ($\triangle  \%$) between species-level average basic wood density from the TWDD (${{\rm{WD}}}_{{\rm{TWDD}}}$), and GWDD^4,9^ and CIRAD^2,26^ datasets (${{\rm{WD}}}_{{\rm{other\; dataset}}}$) (Eq. 2), with the uncertainty estimated as standard deviation.

2 $$\triangle  \% =\frac{{{\rm{WD}}}_{{\rm{TWDD}}}-{{\rm{WD}}}_{{\rm{other\; dataset}}}}{{{\rm{WD}}}_{{\rm{other\; dataset}}}}\ast 100\,[ \% ]$$

## Data Records

The ‘Tervuren xylarium Wood Density Database’ (TWDD) is publicly available on Dryad: 10.5061/dryad.31zcrjf1k. The repository contains three files.

### TWDD.xlsx

The TWDD dataset is stored in an Excel file with the following three sheets:

- ‘Legend’: contains a description of the columns of the dataset.
- ‘TWDD’: contains the wood density data and metadata.
- ‘Overview Table’: contains an overview of the regional geographic and taxonomic coverage of the dataset.

### OvenDryingExperiment.csv

This csv file contains the data of the oven-drying time experiment, with the mass, volume, and density of wood samples in air-dry conditions (columns: m_airdry, V_airdry, and WD_airdry) and after oven-drying for 24 h (columns: m_ovendry_24h, V_ ovendry_24h, and WD_ovendry_24h) and 48 h (columns: m_ovendry_48h, V_ovendry_48h, and WD_ovendry_48h). This was shown for each of the four classes representing low and high wood density and volume (column: Category).

### TWDD_code.R

The R file creates all figures and statistics reported in this paper. To generate a map of the provinces of the Democratic Republic of Congo, we used shapefiles downloaded from Download Democratic Republic of the Congo GIS data. Furthermore, to assess the consistency of our wood density data with other databases, we used the GWDD^4,9^ (10.5061/dryad.234) and CIRAD datasets^2,26^ (10.18167/DVN1/KRVF0E and 10.18167/DVN1/CDHU51).

## Data Overview

The ‘Tervuren xylarium Wood Density Database’ (TWDD) consists of 13,332 wood samples collected in 125 countries across all continents, except Antarctica (Fig. 3A). 72% of the samples in the dataset were collected in Africa (see **Overview Table** in the Excel sheet of the TWDD). In comparison, the GWDD^4,9^ contains only 3,077 African samples and the CIRAD dataset^2,26^ includes 8,798 African samples, accounting for 19% and 37% of the datasets, respectively. The remaining samples were sampled in Asia (22.1%), Oceania (1.5%), South America (1.4%), North America (1.1%), and Europe (0.5%) (Fig. 3A). 57% of all samples were collected in the Democratic Republic of the Congo (DR Congo) (n=7,575) (Fig. 3B)^94–103^.

In terms of taxonomic diversity, the TWDD represents 2,994 tree species and 1,022 genera across 156 plant families (Fig. 3C). In comparison to the GWDD and CIRAD datasets, the TWDD contributes 1,164 new species (39% of all TWDD species), 160 new genera (16% of all TWDD genera), and 8 new plant families (5% of all TWDD families) globally (Table 1). In Africa, the TWDD covers 1,910 tree species across 125 families, with 48% of species, 26% of genera, and 19% of plant families included in the TWDD are newly added, with no prior representation in the GWDD and CIRAD datasets. 31% of all 9,541 recorded Afrotropical tree species^28^ are covered by these three wood density datasets, with the TWDD increasing the taxonomic coverage by 10%. This underscores the TWDD’s crucial role in enhancing global and African taxonomic representation in wood density data to improve the accuracy of biomass carbon and biodiversity assessments across previously data-deficient African forests.

**Table 1:** Newly recorded species, genera, and families compared to the Global Wood Density Database (GWDD)^4,9^ and the CIRAD wood density database^2,26^ at a global and African continental scale.

Region	Taxonomic level	Number of new taxa in TWDD versus GWDD	% of number of new taxa in TWDD versus GWDD	Number of new taxa in TWDD versus CIRAD	% of number of new taxa in TWDD versus CIRAD	Number of new taxa in TWDD versus CIRAD and GWDD	% of number of new taxa in TWDD versus CIRAD and GWDD
Globe	Family	13	8	13	8	8	5
	Genus	288	28	218	21	160	16
	Species	1627	54	1531	51	1164	39
Africa	Family	40	32	28	22	24	19
	Genus	336	49	209	30	176	26
	Species	1369	72	1006	53	920	48

The number of new recorded species, genera, and families is shown compared to other datasets. The percentage (%) of the Tervuren xylarium Wood Density Database (TWDD) with new recorded species, genera, and families is also shown compared to other datasets.

## Technical Validation

### Effect of oven-drying time on oven-dry wood density

**Fig. 4:**
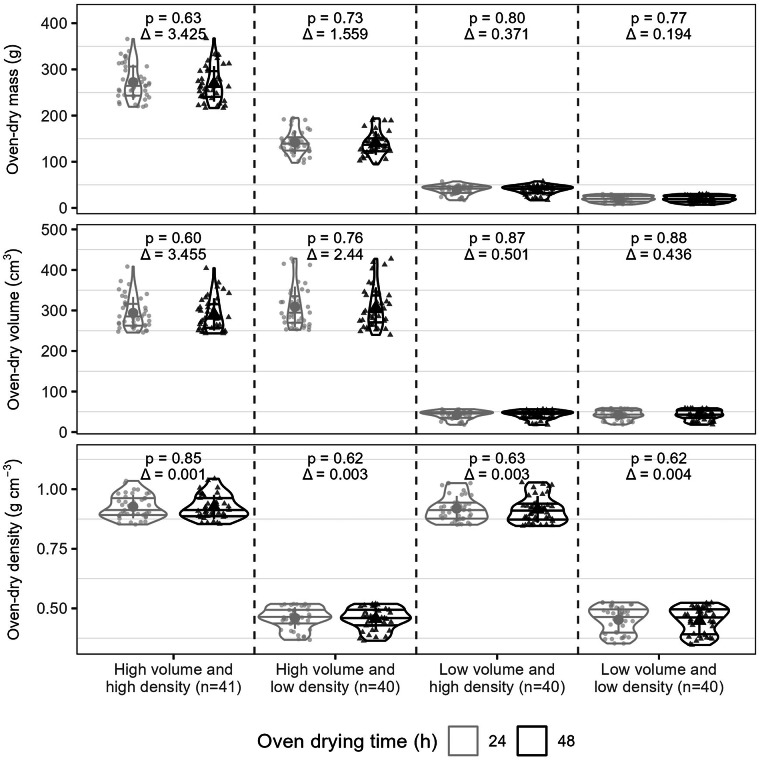
Effect of oven-drying time on oven-dry mass, volume, and density for wood samples with varying volume and density in the Tervuren xylarium. The rows represent the effect of drying wood for 24 h (in grey) and 48 h (in black) at 103 °C on the oven-dry mass (top row, expressed in g), volume (middle row, expressed in cm^3^), and density (bottom row, expressed in g cm^−3^). On the x-axis, the four categories based on the wood volume and density (high versus low) are shown. The sample size n is shown between brackets for each category. The violin plots show the probability distribution with the horizontal lines representing quantiles 25%, 50%, and 75%. The black points show the mean and the vertical lines show the standard deviation. The points represent the individual measurements. The p-value (p) of the Wilcoxon test is shown at the top of each panel, as well as the mean absolute difference between the mass, volume, and density of 24 h versus 48 h oven-drying (Δ).

Our oven-drying experiment comparing 24 h and 48 h oven-drying with a gradual temperature increase cycle from 60 °C to 103 °C showed that there are no significant differences in oven-dry mass, volume, or density across samples of varying volume and density (Wilcoxon test: P > 0.05) (Fig. 4). This suggests that drying during 24 h is sufficient to obtain reliable oven-dry mass and density for wood samples with an air-dry moisture content of approximately 8% (i.e., acclimatized to the Tervuren xylarium’s conditions for more than one year).

Notably, these samples were oven-dried for 1–4 days and subsequently air-dried for several months in the Tervuren xylarium. Therefore, these results do not apply to freshly collected wood. Overall, we recommend first air-drying wood samples, as density measurements are rarely conducted immediately after field collection. Oven-dry wood density can then be determined by oven-drying the air-dried samples at 103 °C for 24 h. To ensure complete drying, we advise checking the mass of a subset of samples (e.g., 5–10) at intervals (e.g., every 6 h) during the drying process, as recommended in international standards (e.g. ISO-13061-1:2014)^51^ and other widely used references^52–56^.

### Conversion factors to compute basic wood density for African tree species

**Fig. 5:**
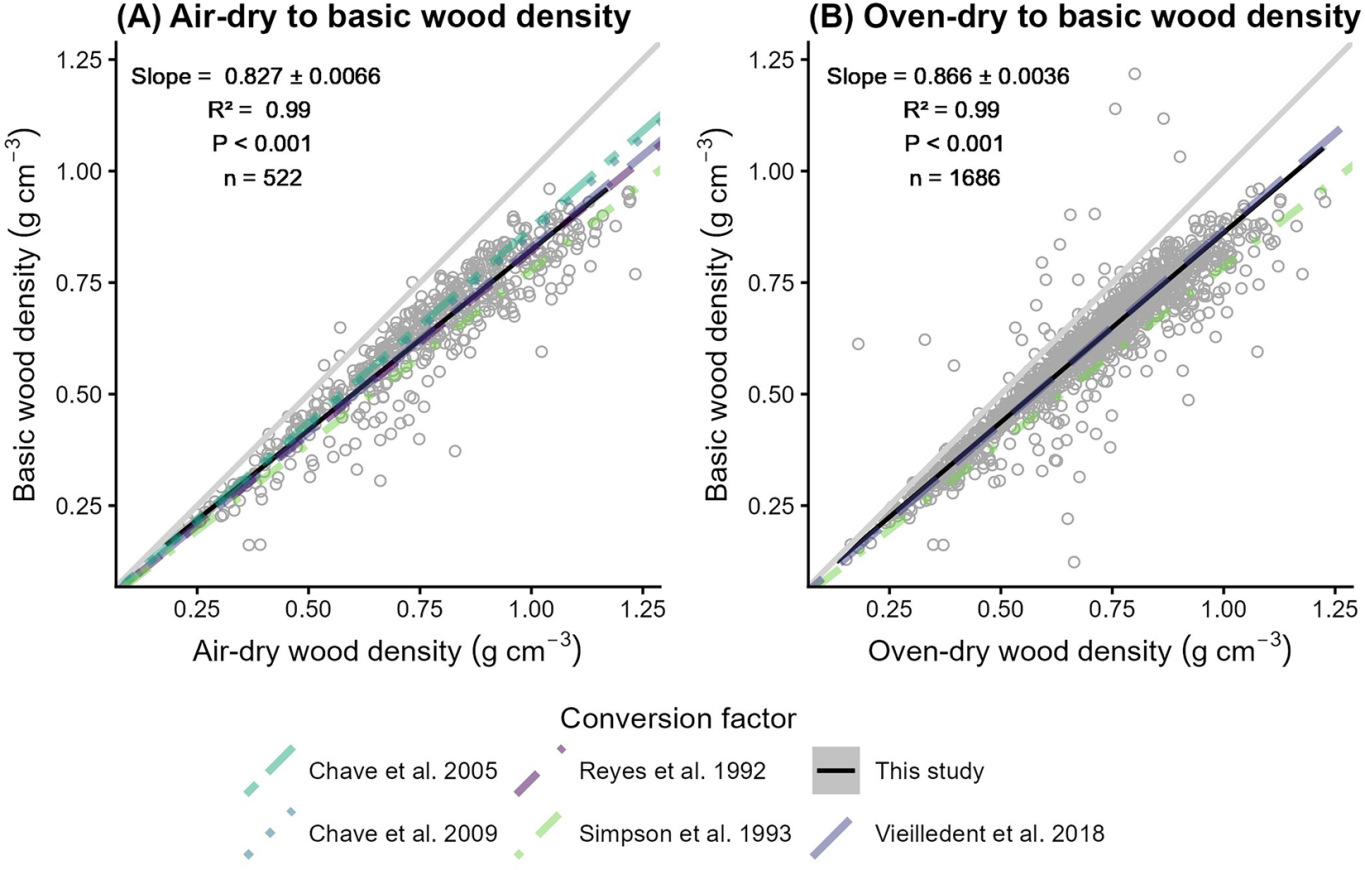
Conversion factors to compute basic wood density from air- and oven-dry wood density for African tree species. (**A**) Relationship between air-dry (x axis) and basic bole wood density (y axis). (**B**) Relationship between oven-dry (x axis) and basic bole wood density (y axis). Dots represent individual wood density measurements for 1,686 samples across five sites in Central Africa, covering 191 tree species, 131 genera, and 41 families. Solid black lines show Major Axis (MA) regression lines, with the 95% confidence interval shown in grey. The slope of the MA regression (i.e., conversion factor), r-squared, P-value, and sample size (n) are shown in the upper left corner. Relationships from other studies are shown including Chave *et al*.^1^ using the formula of Sallenave *et al*.^47^ (slope_air_ = 0.872 at 12% moisture content; dotted line), Chave *et al*.^4^ (slope_air_ = 0.86 at 12% moisture content; two dashed line), Vieilledent *et al*.^2^ (slope_air_ at 12% moisture content = 0.828, slope_oven_ = 0.868; long dashed line), Reyes *et al*.^48^ (slope_air_ = 0.821; dashed line), and Simpson *et al*.^46^ (slope_air_ at 12% moisture content = 0.778, slope_oven_ = 0.785; dot dashed line) (see legend at bottom).

By performing Major Axis regression (MA) on wood density data representing 191 tree species, 131 genera and 41 plant families, we found that the factor to compute basic wood density from air-dry wood density equaled 0.827 ± 0.0066 (R^2^ = 0.99, p < 0.001, n = 522) for African tree species (Fig. 5A). To convert oven-dry wood density to basic wood density, we calculated a factor of 0.866 ± 0.0036 (R^2^ = 0.99, p < 0.001, n = 1,686) (Fig. 5B). Including the intercept in the MA models led to intercepts close to zero for both air-dry (0.016) and oven-dry (0.012) to basic wood density conversions. Conversion factors based on species-level averages of wood density were within the same range as factors using tree-level values for converting air-dry (slope ± standard error: 0.826 ± 0.0104, R^2^ = 0.99, p < 0.001, n = 125) and oven-dry wood density to basic wood density (0.87 ± 0.0067, R^2^ = 1, p < 0.001, n = 191).

Our Africa-specific empirical conversion factors closely aligned with global factors from Vieilledent *et al*.^2^, being 0.14% (or 0.001 g cm^−3^) and 0.24% (or 0.002 g cm^−3^) lower than their air- and oven-dry wood density to basic wood density factors, respectively. Furthermore, the absolute differences in conversion factors between our and other factors were within the range of the error bounds of our models (ranging between 0.004 and 0.007 g cm^−3^). This indicates that conversion factors show minimal geographic variability between Africa and other continents, confirming that this is a global rather than a regional physical relationship. Vieilledent *et al*.^2^ found little difference between conversion factors for tropical and temperate trees. Nonetheless, such conversion factors often exhibit interspecific variation due to differences in wood properties^2^. Therefore, we encourage further research to develop taxon-specific conversion factors by pairing field measurements of green wood density with lab-based oven-dry wood density measurements to test the taxonomical applicability of global conversion factors across continents.

### Comparison of basic wood density with other datasets

For all specimens in the TWDD, we converted oven-dry wood density to basic wood density using the factor from Fig. 5 for African tree species (59.4% of TWDD dataset) and the factor from Vieilledent *et al*.^2^ for specimens sampled elsewhere (28% of TWDD dataset). The median equilibrium air-dry moisture content of wood samples in the TWDD equaled 7.61 ± 0.75% (with interquartile range as uncertainty; n = 6,408). This value was rather low in contrast to ambient air-dry conditions reported in literature^2,49^, frequently ranging between 8 and 18%^2^. However, it was close to the 8% moisture content found in a study on a small subset of the Tervuren xylarium for 118 samples covering 59 species^67^.

The low air-dry moisture content in TWDD illustrates the drawback of using air-dry wood density. Under ambient, temperate climatic conditions^2^, moisture content typically varies between 8 and 18% depending on seasons, geographic regions (e.g., a warmer versus colder climate), storage conditions, and species^2,50,104^. However, here we illustrate that some collections fall outside the average envelope, which further adds uncertainty to global datasets of air-dry wood density. Therefore, instead of relying on air-dry measurements we recommend calculating basic wood density using oven-dry wood density combined with regional (e.g., Fig. 5B for Africa) or global^1,2^ conversion factors. This recommendation of using oven-dry wood density aligns with recent studies^2,4^, while older studies tend towards using air-dry wood density^1,46–48^. Yet, it has been postulated that using oven-dry wood density may underestimate wood density in species with high concentrations of low molecular compounds, which can volatize during oven-drying and reduce oven-dry wood mass^2,105^.

**Fig. 6:**
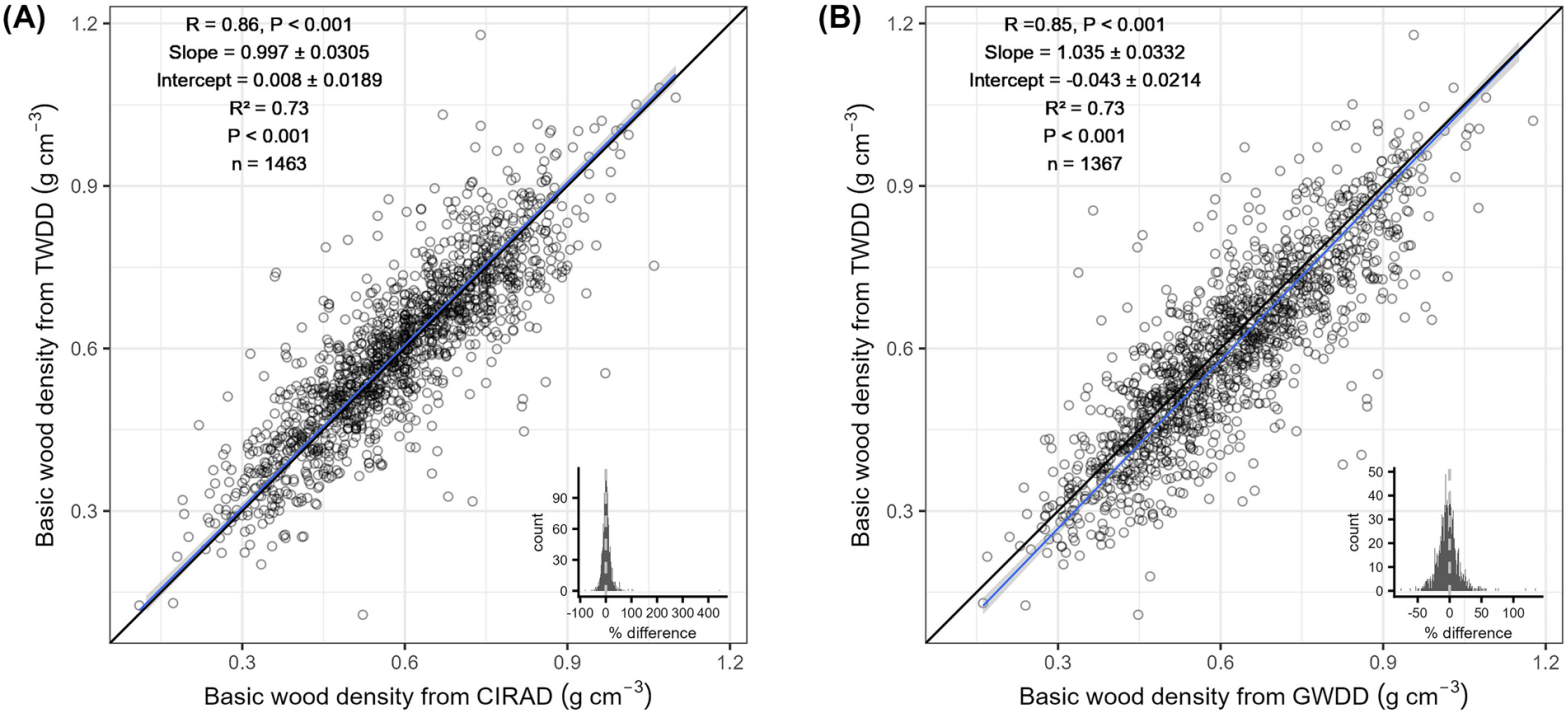
Comparison of species-level basic wood density between Tervuren xylarium Wood Density Database (TWDD) and two other wood density datasets. The Major Axis (MA) regression line between the species-level average of the basic wood density from the Tervuren xylarium Wood Density Database (TWDD) (y-axis), and the CIRAD database^2,26^ (**A**, n = 1,463) and Global Wood Density Database (GWDD)^4,9^ (**B**, n = 1,367) (x-axis) is shown in blue. The Pearson correlation coefficient (R), slope and intercept (with 95% confidence interval) of the MA regression, r-squared, P-value of the overall model, and sample size (n) are shown in the upper left corner. Insets for each panel show histograms of the percentage difference ($\triangle  \%$) in species-level basic wood density between the TWDD (${{\rm{WD}}}_{{\rm{TWDD}}}$) and the other two datasets (${{\rm{WD}}}_{{\rm{other\; dataset}}}$) calculated with Eq. 2. Vertically dashed lines in the histograms inset show the zero line.

To assess consistency between datasets, we compared species-level average basic wood densities from the TWDD with the GWDD^4,9^ and CIRAD datasets^2,26^. By calculating the Pearson correlation coefficient (R) and performing MA regression, we showed that species-level basic wood densities of the TWDD were similar to the CIRAD (Fig. 6A) (R = 0.86, p < 0.001; MA regression: R^2^ = 0.73, p < 0.001; n = 1,463) and GWDD datasets (Fig. 6B) (R = 0.85, p < 0.001; R^2^ = 0.73, p < 0.001; n = 1,367). Furthermore, using a Wilcoxon test, we found that there was a significant but small difference between our species-level average basic wood densities and estimates from GWDD (Fig. 6B inset) (average percentage difference ± standard deviation: −2.7 ± 16.6%; Wilcoxon test: p < 0.01). By contrast, no significant difference was observed between species-level average basic wood densities from TWDD and CIRAD (Fig. 6A inset) (2.6 ± 19.6%; p = 0.28). The Root Mean Squared (RMSE) equaled 0.0956 and 0.0843 g cm^−3^ for the GWDD and CIRAD datasets, respectively.

There are five potential reasons for species-level mismatches between the TWDD and the two other datasets. First, differences in oven-drying time and temperature might explain the difference between the datasets (see above). Second, previous datasets partly consisted of basic wood density data based on oven-dry and green field measurements (about 40% of GWDD and 17% of CIRAD), as well as converted air-dry densities (about 60% of GWDD and 83% of CIRAD). The high variability of moisture content of wood samples in air-dry conditions as well as inconsistencies in previously used conversion factors^1,2,4,46–48^ might have led to inaccurate basic wood density estimates in other datasets. Third, intraspecific variation in wood density might lead to differences between datasets due to low sample size for certain species.

Fourth, 18% of the TWDD was based on branches instead of wood collected from the tree stem. Consequently, wood density is most likely underestimated for some wood samples since wood density of branches is often lower than stem wood density^106,107^, particularly in dense wooded species^108^. Branches are predominantly sapwood, lacking the extractives that make stem heartwood heavier^109^.

Finally, the volume, type (i.e., pith-to-bark cores, disks, and heart- versus sapwood samples), collection height type (e.g., branch or bole), and age of the sampled wood influence wood density estimates^110,111^.

## Data Availability

All data is available on Dryad: 10.5061/dryad.31zcrjf1k^112^.

## Code availability

All code is available on Dryad: 10.5061/dryad.31zcrjf1k. The code reproduces all the figures and tables in this manuscript^112^.
